# Natural history of hypophosphatasia in Chinese patients younger than 18 years: a single-center retrospective observational study

**DOI:** 10.3389/fendo.2026.1941377

**Published:** 2026-09-17

**Authors:** Jingjie Luo, Min Liu, Xiaoya Ren, Meijuan Liu, Jiajia Chen, Xinmeng Wang, Zheng Yuan, Bingyan Cao, Chunxiu Gong

**Affiliations:** 1 Department of Endocrinology, Genetics and Metabolism, Beijing Children’s Hospital, Capital Medical University, National Center for Children’s Health, Beijing, China; 2 Department of Endocrinology, Capital Center for Children’s Health, Capital Medical University, Beijing, China

**Keywords:** ALPL, genotype-phenotype correlation, hypophosphatasia, natural history, pediatric

## Abstract

**Purpose:**

To summarize the clinical features, natural history, and ALPL variant spectrum of Chinese children with hypophosphatasia (HPP), and to evaluate diagnostic features across clinical subgroups.

**Methods:**

This single-center retrospective observational study included children diagnosed with hypophosphatasia at our hospital who had not received enzyme replacement therapy. Published Chinese pediatric hypophosphatasia cases were also reviewed and combined with the single-center cohort for descriptive analyses.

**Results:**

Ninety-two children were included in the pooled cohort, comprising 23 patients in the single-center cohort and 69 cases identified from the literature. Baseline features were broadly comparable between single-center cohort and the literature cohort (P > 0.05). In the pooled cohort, there were 34 severe HPP (including 6 perinatal hypophosphatasia and 28 infantile hypophosphatasia), 34 childhood HPP, and 24 odonto-HPP. Median ages at onset were 0.09, 1.13, and 1.45 years, and median diagnostic delays were 0.15, 3.00, and 1.44 years, respectively. Height and weight Z-scores were lowest in severe HPP and least affected in odonto-HPP (all P < 0.001). Severe HPP had lower alkaline phosphatase, phosphate, and parathyroid hormone levels and higher calcium levels than the other groups. Among 70 genetically tested patients, 85 ALPL variants were identified; compound heterozygosity (78.6%) and missense variants (72.9%) predominated. In our single-center cohort, 3/8 severe HPP patients died, 4/9 childhood HPP patients progressed, and all 6 odonto-HPP patients remained stable during follow-up.

**Conclusion:**

Chinese pediatric hypophosphatasia is clinically and genetically heterogeneous. Earlier onset was associated with more severe biochemical abnormalities, impaired growth, and poorer outcomes. Childhood hypophosphatasia was prone to diagnostic delay and did not show spontaneous resolution. Atraumatic early loss of primary teeth and persistently low age- and sex-adjusted alkaline phosphatase are key to early recognition. However, larger prospective studies are required to confirm these observations.

## 1 Introduction

Hypophosphatasia (HPP) is an inherited metabolic bone disease caused by loss-of-function variants in ALPL, which encodes tissue-nonspecific alkaline phosphatase (TNSALP). TNSALP is highly expressed in bone, teeth, liver, and kidneys and hydrolyzes extracellular inorganic pyrophosphate (PPi) into inorganic phosphate (Pi), thereby promoting skeletal and dental mineralization. Deficient TNSALP activity causes PPi accumulation, impaired hydroxyapatite formation, defective mineralization, and dental abnormalities. Hypophosphatasia was first described by the Canadian physician John C. Rathbun in 1948 and was included in China’s First List of Rare Diseases by the National Health Commission of China in 2018. The clinical phenotypic spectrum of hypophosphatasia is broad, ranging from perinatal lethal hypophosphatasia to odontohypophosphatasia presenting only with premature loss of primary teeth. Approximately 50% of patients with infantile hypophosphatasia who did not receive enzyme replacement therapy died during infancy, primarily due to severe respiratory complications (1, 2).

Severe perinatal and infantile hypophosphatasia can be associated with respiratory failure, hypercalcemia, seizures, and early death (1, 3). In contrast, childhood hypophosphatasia may present with nonspecific dental, skeletal, motor, or growth problems and can therefore be missed or misdiagnosed (4). Natural history data have been reported in several international cohorts (5–10), but clinical and genetic data from Chinese children remain limited.

This study aimed to describe the natural history and ALPL variant spectrum of pediatric hypophosphatasia in Chinese children, with emphasis on clinical features, genotype-phenotype associations, longitudinal outcomes, and factors that may improve early recognition and reduce diagnostic delay (11).

## 2 Methods

### 2.1 Participants and case review

We performed a single-center observational follow-up study and a literature-based review of Chinese pediatric hypophosphatasia cases. The single-center cohort included patients first diagnosed with hypophosphatasia at our hospital before April 30, 2026. Inclusion criteria were age < 18 years and a diagnosis of hypophosphatasia according to the 2024 pediatric diagnostic criteria proposed by the HPP International Working Group (12), and availability of clinical data obtained before exposure to enzyme replacement therapy (ERT). Patients were either treatment-naïve to ERT or contributed only data collected before ERT initiation. Exclusion criteria were coexisting metabolic bone disease or incomplete clinical data. The study was approved by the Ethics Committee of Beijing Children’s Hospital, Capital Medical University (approval no. [2026]-E-023-R). Written informed consent was obtained from all patients and their parents or legal guardians before participation.

For the literature review, PubMed was searched using (hypophosphatasia[Title/Abstract]) AND China. CNKI, VIP, Wanfang, and the Chinese Medical Journal Full-text Database were searched using terms equivalent to hypophosphatasia in title/abstract. The search covered each database from inception to December 2025. Exclusion criteria included articles unrelated to hypophosphatasia, reports of patients aged ≥18 years, studies other than cohorts or case reports, cases treated with enzyme replacement therapy, cases for which the full text or required case-level data were unavailable, and duplicate or overlapping publications, with only the most recent report from the same research group retained. Patients from the literature review were combined with the single-center patients to form the pooled cohort.

### 2.2 Data collection

For the single-center cohort, clinical data were obtained from electronic and paper medical records. For literature cases, data were extracted independently from the published reports. Variables included sex, age at onset, age at diagnosis, family history, height, weight, skeletal findings, dental manifestations, motor development, neurological manifestations, serum alkaline phosphatase (ALP), calcium, phosphate, parathyroid hormone (PTH), 25-hydroxyvitamin D, radiographs, renal ultrasound findings, genetic results, and follow-up status.

Age at onset was defined as the age at which the first hypophosphatasia-related clinical manifestation was documented. Diagnostic delay was defined as age at diagnosis minus age at onset. Height-for-age Z-score (HAZ) and weight-for-age Z-score (WAZ) were calculated using the 2009 Chinese reference standards for children aged 0–18 years (13). Short stature was defined as HAZ < -2 SD (14). Disease progression was defined as new symptoms within the previous 2 years or a Radiographic Global Impression of Change (RGI-C) score ≤-2 on wrist or knee radiographs. Disease stability was defined as no new symptoms and an RGI-C score between -1 and 1. Improvement was defined as no new symptoms and an RGI-C score ≥ 2.

### 2.3 Genetic analysis

Genomic DNA was extracted from 3 mL of EDTA-anticoagulated peripheral blood from patients and their parents. Cases diagnosed before 2015 were tested by Sanger sequencing; cases diagnosed from 2015 onward were tested by next-generation sequencing. Candidate variants were confirmed by Sanger sequencing, and parental samples were analyzed to determine variant origin. Variants absent from ALPL Gene Variant Database (https://alplmutationdatabase.jku.at/), ClinVar (http://www.ncbi.nlm.nih.gov/clinvar/) and previously published literature were defined as previously unreported variants.

### 2.4 Statistical analysis

Data processing and analysis were performed using R version 4.3.3. Normally distributed continuous variables are presented as mean ± SD and were compared using independent-sample t tests for two groups or one-way analysis of variance for multiple groups. Non-normally distributed variables are presented as median (Q1, Q3) and were compared using the Mann-Whitney U test for two groups or the Kruskal-Wallis H test for multiple groups. Categorical variables are presented as n (%) and were compared using the chi-square test or Fisher’s exact test as appropriate. Two-sided P < 0.05 was considered statistically significant.

## 3 Results

### 3.1 Patient characteristics

The final pooled cohort included 92 Chinese children with hypophosphatasia, comprising 23 patients diagnosed at our center and 69 patients extracted from 39 published reports. The literature screening process is shown in **Figure 1**. Clinical characteristics are shown in **Table 1**. Most baseline variables were comparable between the single-center cohort and the literature cohort (P^c^ > 0.05), except for the clinical symptoms and the prognosis part which have missing data in the literature cohort. Although hypophosphatasia has commonly been classified into six to seven clinical types in previous studies, patients in the present study were grouped into three major categories for comparative analysis. Patients were grouped into three categories: severe HPP, onset at ≤6 months of age, including perinatal and infantile hypophosphatasia; childhood HPP, onset from >6 months to <18 years with skeletal manifestations; and odonto-HPP, defined as dental manifestations only.

**Figure 1:**
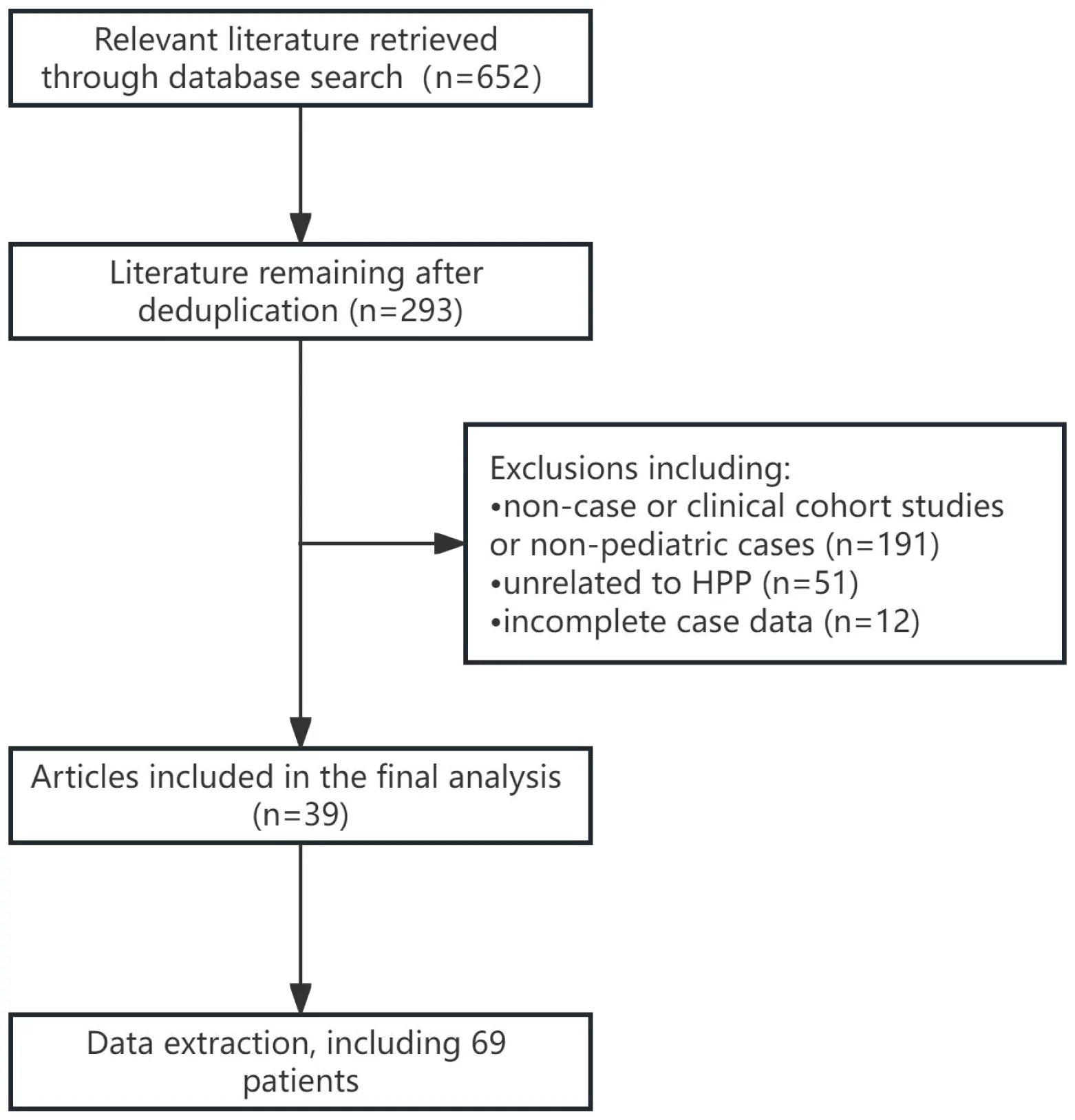
Literature screening flowchart and results. The searched databases and retrieved records were PubMed (n = 59), CNKI (n = 124), VIP (n = 140), Wanfang (n = 307), and Chinese Medical Journal Full-text Database (n = 22).

**Table 1:** Characteristics of pediatric patients with hypophosphatasia.

Variables	Pooled cohort						Single-center cohort						P^c^
	Total (n = 92)	Severe (n = 34)	Childhood (n = 34)	odonto (n = 24)	Statistic	P^a^	Total (n = 23)	Severe (n = 8)	Childhood (n = 9)	odonto (n = 6)	Statistic	P^b^	
Sex, n (%)^d^					-	0.603					-	0.432	0.851
Male	59 (64.13)d	19 (55.88)	24 (70.59)	16 (66.67)			14 (60.87)	4 (50.00)	7 (77.78)	3 (50.00)			
Female	32 (34.78)	14 (41.18)	10 (29.41)	8 (33.33)			9 (39.13)	4 (50.00)	2 (22.22)	3 (50.00)			
Age at symptom onset (years)	1.00 (0.10, 1.43)	0.09 (0.00,0.20)	1.13 (1.00,1.50)	1.45 (1.00,2.00)	χ² = 46.14#	<.001	1.00 (0.25, 1.38)	0.13 (0.06,0.25)	1.17 (1.00,1.36)	1.66 (1.27,1.94)	χ² = 16.42#	<.001	0.419
Age at diagnosis (years)	1.87 (0.42, 5.00)	0.32 (0.17,0.45)	4.00 (2.33,7.00)	2.92 (1.75,5.00)	χ² = 43.78#	<.001	1.84 (0.51, 3.65)	0.29 (0.23,0.50)	3.47 (1.84,6.83)	3.26 (2.17,4.75)	χ² = 15.04#	<.001	0.987
Diagnostic delay (years)	1.00 (0.15, 3.00)	0.15 (0.08,0.29)	3.00 (1.04,7.00)	1.44 (1.00,4.00)	χ² = 20.58#	<.001	0.48 (0.16, 2.57)	0.22 (0.09,0.25)	2.30 (0.58,5.43)	1.68 (0.45,3.62)	χ² = 7.79#	0.020	0.763
Height-for-age Z-score	-2.00 ± 2.07	-3.18 ± 1.63	-2.03 ± 1.89	0.11 ± 1.26	F = 19.02	<.001	-1.89 ± 2.11	-2.94 ± 1.76	-2.33 ± 1.90	0.10 ± 1.61	F = 5.36	0.014	0.762
Weight-for-age Z-score	-1.52 ± 2.15	-2.91 ± 1.84	-0.87 ± 1.63	0.36 ± 1.39	F = 19.15	<.001	-1.29 ± 2.24	-2.99 ± 1.43	-0.84 ± 2.17	0.31 ± 1.92	F = 5.74	0.011	0.505
Serum ALP (U/L)	27.00 (12.00, 41.00)	9.00 (7.00,25.00)	33.00 (27.00,45.50)	31.50 (22.00,58.75)	χ² = 27.81#	<.001	37.57 ± 21.63	28.25 ± 23.24	40.67 ± 8.54	45.33 ± 30.93	F = 1.25	0.308	0.038
Serum calcium (mmol/L)	2.63 (2.45, 3.27)	3.11 (2.80,3.55)	2.48 (2.39,2.67)	2.53 (2.45,2.55)	χ² = 15.49#	<.001	2.50 (2.42, 3.04)	3.35 (3.08,3.70)	2.45 (2.39,2.50)	2.45 (2.40,2.52)	χ² = 9.27#	0.010	0.075
Serum phosphate (mmol/L)	1.89 (1.65, 2.08)	1.58 (1.31,1.88)	1.96 (1.80,2.09)	1.99 (1.84,2.11)	χ² = 10.26#	0.006	1.89 (1.65, 2.05)	1.39 (1.16,1.65)	2.08 (1.90,2.11)	1.92 (1.74,1.95)	χ² = 14.52#	<.001	0.484
PTH (pg/mL)	12.70 (3.00, 22.77)	3.00 (1.65,12.80)	14.60 (9.84,22.94)	22.40 (12.03,27.38)	χ² = 12.97#	0.002	15.46 ± 12.49	3.70 ± 4.48	21.50 ± 10.34	27.07 ± 5.55	F = 16.22	<.001	0.930
Serum creatinine (µmol/L)	30.97 ± 8.62	35.31 ± 8.98	27.50 ± 4.28	27.00 ± 8.57	F = 3.36	0.052	30.54 ± 8.92	35.60 ± 10.10	27.50 ± 4.28	27.33 ± 9.34	F = 2.36	0.123	0.611
Early loss of primary teeth, n (%)^e^					-	<.001					-	<.001	0.747
–	30 (32.61)	28 (82.35)	2 (5.88)	0 (0.00)			7 (30.43)	7 (87.50)	0 (0.00)	0 (0.00)			
+	59 (64.13)	5 (14.71)	30 (88.24)	24 (100.00)			16 (69.57)	1 (12.50)	9 (100.00)	6 (100.00)			
Short stature, n (%)^e^					-	<.001					-	0.250	0.128
–	47 (51.09)	13 (38.24)	14 (41.18)	20 (83.33)			12 (52.17)	3 (37.50)	4 (44.44)	5 (83.33)			
+	35 (38.04)	20 (58.82)	13 (38.24)	2 (8.33)			11 (47.83)	5 (62.50)	5 (55.56)	1 (16.67)			
Delayed motor milestones, n (%)^e^					χ² = 36.02	<.001					-	<.001	<.001
–	30 (32.61)	10 (29.41)	4 (11.76)	16 (66.67)			7 (30.43)	0 (0.00)	1 (11.11)	6 (100.00)			
+	32 (34.78)	20 (58.82)	12 (35.29)	0 (0.00)			16 (69.57)	8 (100.00)	8 (88.89)	0 (0.00)			
Chronic musculoskeletal pain, n (%)^e^					χ² = 28.11	<.001					-	0.009	0.006
–	54 (58.70)	26 (76.47)	9 (26.47)	19 (79.17)			18 (78.26)	8 (100.00)	4 (44.44)	6 (100.00)			
+	15 (16.30)	2 (5.88)	13 (38.24)	0 (0.00)			5 (21.74)	0 (0.00)	5 (55.56)	0 (0.00)			
Impaired mobility, n (%)e					χ² = 41.88	<.001					-	<.001	<.001
–	25 (27.17)	7 (20.59)	1 (2.94)	17 (70.83)			7 (30.43)	1 (12.50)	0 (0.00)	6 (100.00)			
+	37 (40.22)	20 (58.82)	17 (50.00)	0 (0.00)			16 (69.57)	7 (87.50)	9 (100.00)	0 (0.00)			
Genu valgum/varum, n (%)^e^					χ² = 20.65	<.001					-	0.032	<.001
–	45 (48.91)	22 (64.71)	7 (20.59)	16 (66.67)			15 (65.22)	6 (75.00)	3 (33.33)	6 (100.00)			
+	15 (16.30)	5 (14.71)	10 (29.41)	0 (0.00)			8 (34.78)	2 (25.00)	6 (66.67)	0 (0.00)			
Craniosynostosis, n (%)^e^					-	0.002					-	0.002	<.001
–	52 (56.52)	22 (64.71)	14 (41.18)	16 (66.67)			18 (78.26)	3 (37.50)	9 (100.00)	6 (100.00)			
+	5 (5.43)	5 (14.71)	0 (0.00)	0 (0.00)			5 (21.74)	5 (62.50)	0 (0.00)	0 (0.00)			
Failure to thrive, n (%)^e^					χ² = 41.34	<.001					-	<.001	<.001
–	39 (42.39)	9 (26.47)	14 (41.18)	16 (66.67)			15 (65.22)	1 (12.50)	8 (88.89)	6 (100.00)			
+	20 (21.74)	19 (55.88)	1 (2.94)	0 (0.00)			8 (34.78)	7 (87.50)	1 (11.11)	0 (0.00)			
Recurrent fractures, n (%)^e^					-	<.001					-	0.090	0.001
–	53 (57.61)	24 (70.59)	10 (29.41)	19 (79.17)			20 (86.96)	8 (100.00)	6 (66.67)	6 (100.00)			
+	12 (13.04)	4 (11.76)	8 (23.53)	0 (0.00)			3 (13.04)	0 (0.00)	3 (33.33)	0 (0.00)			
Recurrent pneumonia, n (%)^e^					-	<.001					-	0.609	<.001
–	50 (54.35)	19 (55.88)	15 (44.12)	16 (66.67)			22 (95.65)	7 (87.50)	9 (100.00)	6 (100.00)			
+	8 (8.70)	8 (23.53)	0 (0.00)	0 (0.00)			1 (4.35)	1 (12.50)	0 (0.00)	0 (0.00)			
Hypotonia, n (%)^e^					χ² = 28.47	<.001					-	0.038	<.001
–	34 (36.96)	9 (26.47)	9 (26.47)	16 (66.67)			11 (47.83)	1 (12.50)	5 (55.56)	5 (83.33)			
+	27 (29.35)	19 (55.88)	7 (20.59)	1 (4.17)			12 (52.17)	7 (87.50)	4 (44.44)	1 (16.67)			
Vitamin B6-responsive seizures, n (%)^e^					-	0.002					-	0.609	<.001
–	59 (64.13)	26 (76.47)	17 (50.00)	16 (66.67)			22 (95.65)	7 (87.50)	9 (100.00)	6 (100.00)			
+	4 (4.35)	4 (11.76)	0 (0.00)	0 (0.00)			1 (4.35)	1 (12.50)	0 (0.00)	0 (0.00)			
Nephrocalcinosis, n (%)^e^					-	<.001					-	<.001	<.001
Not examined	58 (63.04)	16 (47.06)	25 (73.53)	17 (70.83)			4 (17.39)	0 (0.00)	2 (22.22)	2 (33.33)			
–	16 (17.39)	2 (5.88)	7 (20.59)	7 (29.17)			9 (39.13)	0 (0.00)	5 (55.56)	4 (66.67)			
+	18 (19.57)	16 (47.06)	2 (5.88)	0 (0.00)			10 (43.48)	8 (100.00)	2 (22.22)	0 (0.00)			
Radiographic abnormalities, n (%)^e^					-	<.001					-	0.005	0.012
–	13 (14.13)	1 (2.94)	0 (0.00)	12 (50.00)			5 (21.74)	1 (12.50)	0 (0.00)	4 (66.67)			
+	59 (64.13)	33 (97.06)	22 (64.71)	4 (16.67)			18 (78.26)	7 (87.50)	9 (100.00)	2 (33.33)			
ALPL variant detected, n (%)					χ² = 3.05	0.217					-	0.449	0.397
Not examined	22 (23.91)	11 (32.35)	8 (23.53)	3 (12.50)			7 (30.43)	4 (50.00)	2 (22.22)	1 (16.67)			
Positive	70 (76.09)	23 (67.65)	26 (76.47)	21 (87.50)			16 (69.57)	4 (50.00)	7 (77.78)	5 (83.33)			
Genotype, n (%)	n = 70	n = 23	n = 26	n = 21	-	0.404	n = 16	n = 4	n = 7	n = 5	-	0.261	0.877
Homozygous	4 (5.71)	2 (8.70)	2 (7.69)	0 (0.00)			1 (6.25)	0 (0.00)	1 (14.29)	0 (0.00)			
Heterozygous	11 (15.71)	2 (8.70)	5 (19.23)	4 (19.05)			2 (12.5)	0 (0.00)	0 (0.00)	2 (60.00)			
Compound heterozygous	55 (78.57)	19 (82.60)	19 (73.08)	17 (80.95)			13 (81.25)	4 (100.00)	6 (85.71)	3 (40.00)			
Outcome, n (%)					X² = 38.93	<.001					-	0.001	<.001
Lost to follow-up	42 (45.65)	10 (29.41)	20 (58.82)	12 (50.00)			3 (13.04)	3 (37.50)	0 (0.00)	0 (0.00)			
Death	18 (19.57)	18 (52.94)	0 (0.00)	0 (0.00)			3 (13.04)	3 (37.50)	0 (0.00)	0 (0.00)			
Alive	32 (34.78)	6 (17.65)	14 (41.18)	12 (50.00)			17 (73.91)	2 (25.00)	9 (100.00)	6 (100.00)			

F, analysis of variance; ^#^Kruskal-Wallis test; χ², chi-square test; -, Fisher’s exact test; SD, standard deviation; M, median; Q1, first quartile; Q3, third quartile; ALP, alkaline phosphatase; PTH, parathyroid hormone; +, positive; -, negative. P^a^compares the severe HPP, childhood HPP and odonto-HPP groups within the pooled cohort; P^b^compares the three HPP groups within the single-center cohort; P^c^compares the single-center cohort with the literature cohort. ^d^One perinatal lethal case had unspecified sex in literature review cohorts. ^e^Some data were missing; therefore, percentages were calculated using the observed number divided by the total number of patients in the relevant cohort or group. Bolded entries indicate the item names of categorical variables. Bolding is used for ease of reading.

In the pooled cohort, median ages at symptom onset were 0.09, 1.13, and 1.45 years in the severe, childhood, odonto-HPP, respectively. Median ages at diagnosis were 0.32, 4.00, and 2.92 years, and median diagnostic delays were 0.15, 3.00, and 1.44 years, respectively (all P < 0.001). Overall HAZ was -2.00 ± 2.07 and WAZ was -1.52 ± 2.15. Growth impairment was greatest in severe HPP and mildest in odonto-HPP: HAZ was -3.18 ± 1.63, -2.03 ± 1.89, and 0.11 ± 1.26, and WAZ was -2.91 ± 1.84, -0.87 ± 1.63, and 0.36 ± 1.39 in the severe, childhood, odonto-HPP, respectively (all P < 0.001).

ALP, serum calcium, phosphate, and PTH differed significantly among groups (all P < 0.05). Severe HPP had the highest calcium level [3.11 (2.80, 3.55) mmol/L] and the lowest ALP [9.00 (7.00, 25.00) U/L], phosphate [1.58 (1.31, 1.88) mmol/L], and PTH [3.00 (1.65, 12.80) pg/mL]. Childhood and odonto-HPP had relatively higher ALP, phosphate, and PTH levels and lower calcium levels. The single-center cohort showed a similar pattern (**Table 1**).

Because symptom and outcome data were incomplete in the literature cohort, longitudinal assessment focused on single-center cohort, which had not received disease-specific therapy. Phenotypes differed substantially across the three groups. In severe HPP, delayed motor milestones and nephrocalcinosis were present in all patients, and failure to thrive, hypotonia, and impaired mobility each occurred in 87.50%. In childhood HPP, early loss of primary teeth (100%), impaired mobility (100%), delayed motor milestones (88.89%), and short stature (55.56%) were prominent. In odonto-HPP, early loss of primary teeth or dental caries was the dominant manifestation (100%).

### 3.2 Genetic findings

All genetically tested patients in the pooled cohort had ALPL variants. In total, 70/92 patients underwent genetic testing, and 85 ALPL variant sites were identified (**Figure 2**). Compound heterozygous variants were the most common genotype (78.6%), followed by heterozygous (15.7%) and homozygous variants (5.7%). Missense variants were the predominant variant type (62/85, 72.9%), followed by frameshift variants (8/85, 9.4%), splice-site variants (4/85, 4.7%), and in-frame insertions/deletions (4/85, 4.7%).

**Figure 2:**
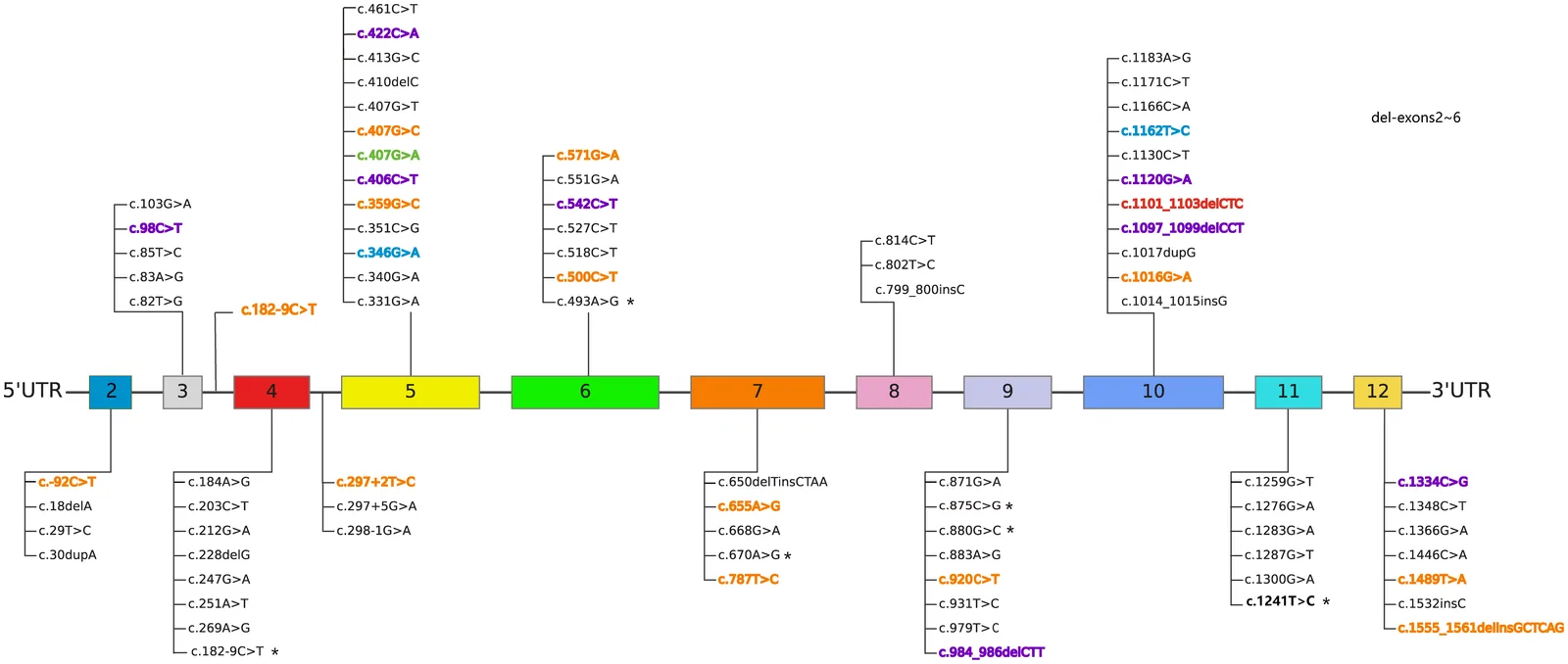
ALPL variants identified in Chinese pediatric patients with hypophosphatasia. Colors indicate recurrence frequency: green, 10; red, 5; blue, 4; purple, 3; orange, 2; black, 1. Asterisks indicate previously unreported variants.

The most frequent variant was c.407G>A in exon 5 (10/140 alleles, 7.14%), which was observed in all three groups but was more common in odontohypophosphatasia (severe: childhood: odonto-HPP = 1:3:6). The c.1101_1103delCTC variant in exon 10 and c.346G>A in exon 5 each accounted for 5/140 alleles (3.57%); the former was found only in severe HPP, whereas the latter was observed in all three groups. The c.1162T>C variant in exon 10 (4/140 alleles, 2.86%) was found only in childhood and odonto-HPP. Five previously unreported variants were identified in the single-center cohort: c.875C>G, c.182-9C>T, c.880G>C, c.493A>G, and c.1241T>C.

### 3.3 Clinical manifestations and natural history

The natural history of 23 patients in the single-center cohort without disease-specific therapy is shown in **Figure 3**. Mean follow-up duration was 3.3 years (range, 0–11 years). All eight patients in severe HPP presented initially with failure to thrive and had nephrocalcinosis at first evaluation. One patient in severe HPP (PA-7) had a neonatal seizure and survived to 7.4 years; at the latest follow-up, the remaining manifestations were atraumatic tooth loss and slow linear growth.

**Figure 3:**
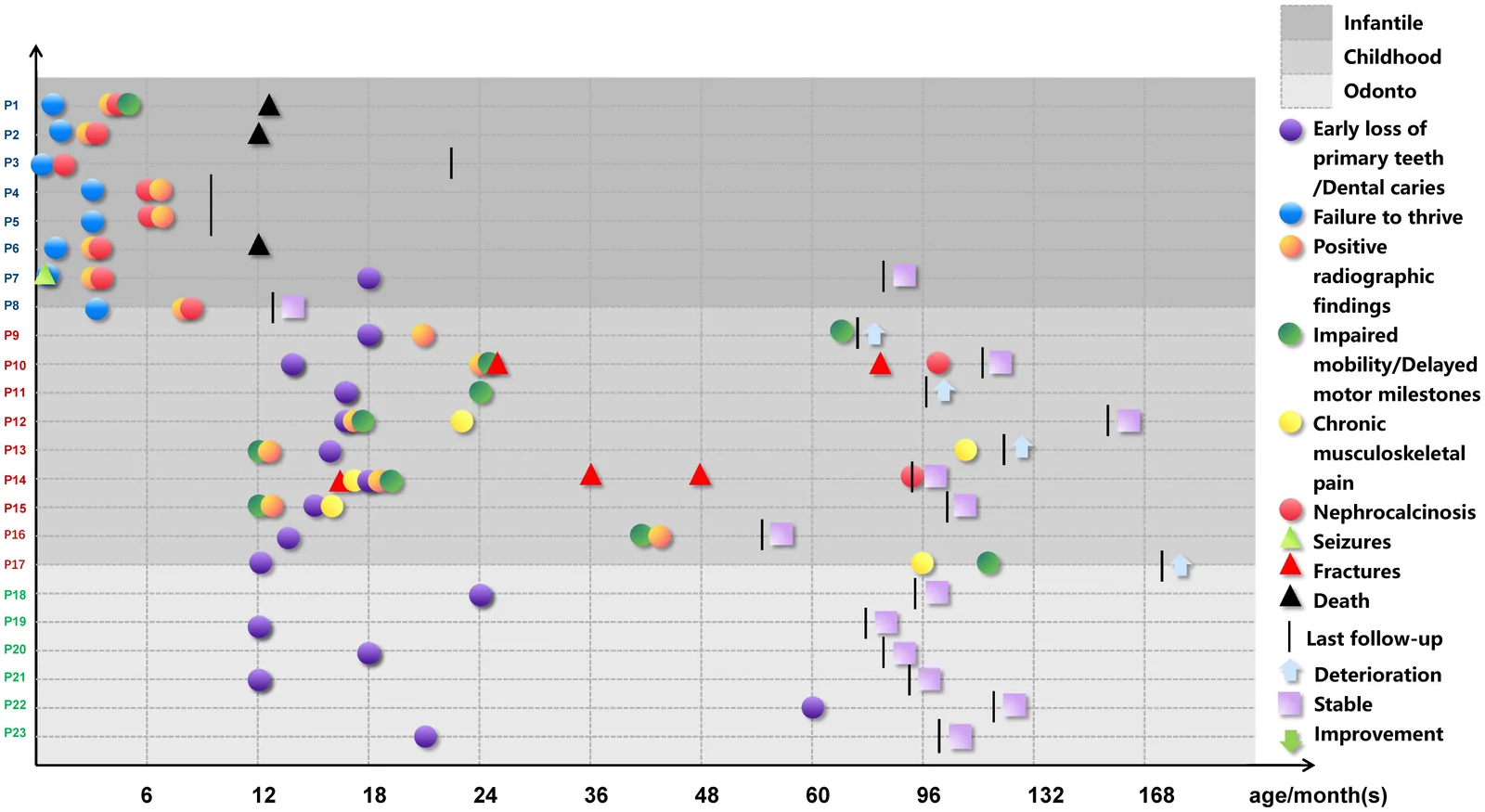
Natural history timeline of 23 pediatric patients with hypophosphatasia. The x-axis indicates age in months, and each row represents one patient.

All patients in childhood and odonto-HPP developed early loss of primary teeth or severe dental caries at 1–2 years of age. In childhood HPP, the initial manifestation was early primary tooth loss in 6/9 patients, impaired mobility in 2/9, and fracture after minor trauma in 1/9. Impaired mobility occurred in all childhood HPP patients, genu valgum or varum in 6/9, chronic musculoskeletal pain in 5/9, recurrent fractures after minor trauma in PA-10 and PA-14, and nephrocalcinosis in 2/9.

At the latest follow-up, three patients in severe HPP (PA-1, PA-2, and PA-6) had died at approximately 12–13 months due to recurrent pneumonia, pleural effusion, or respiratory failure; PA-3, PA-4, and PA-5 were lost to follow-up. PA-7 and PA-8 were followed to 7.4 and 1.1 years, respectively, and neither required respiratory support. In childhood HPP, latest follow-up ages were 4.7–14.9 years; 4/9 patients progressed and 5/9 remained stable, with no spontaneous improvement. All six patients in odonto-HPP were stable at latest follow-up (age, 6.3–10 years).

### 3.4 Representative cases

PA-7 was a 7-year-old girl referred for poor growth for 7 years and tooth loss for 6 years. She developed failure to thrive, nausea, and one afebrile seizure after birth. Hypercalcemia (5 mmol/L) was identified at a local hospital and treated symptomatically. At our hospital, ALP was 72 U/L, calcium was 2.9 mmol/L, and PTH was 1.3 pg/mL. Renal tubular acidosis was considered, and potassium citrate was administered for 1 month without further follow-up. Atraumatic loss of primary teeth began 6 years before the current presentation. She was born at term by vaginal delivery (birth weight 3.3 kg; birth length 49 cm), walked at 1 year, and could run and jump at 3–4 years.

At presentation, height was 115.5 cm (3rd-10th percentile), weight was 21.6 kg (25th percentile), and physical examination showed wide intercanthal distance, four upper teeth and three lower teeth, normal gait, mild rib flaring, and no wrist or ankle widening. Serum ALP was low (40 U/L), phosphate was high (1.94 mmol/L), and calcium, 25-hydroxyvitamin D, and urinary calcium/creatinine ratio were normal. Radiographs showed patchy reductions in metaphyseal bone density in long bones (**Figures 4A, B, D, E**). Abdominal ultrasound showed bilateral renal medullary calcification. Whole-exome sequencing identified compound heterozygous ALPL variants: c.1120G>A (p.Val374Met), inherited from her father, and c.875C>G (p.Pro292Arg), inherited from her mother. The diagnosis was revised to hypophosphatasia. PA-7 was the only neonatally symptomatic patient in our follow-up cohort who survived to school age.

**Figure 4:**
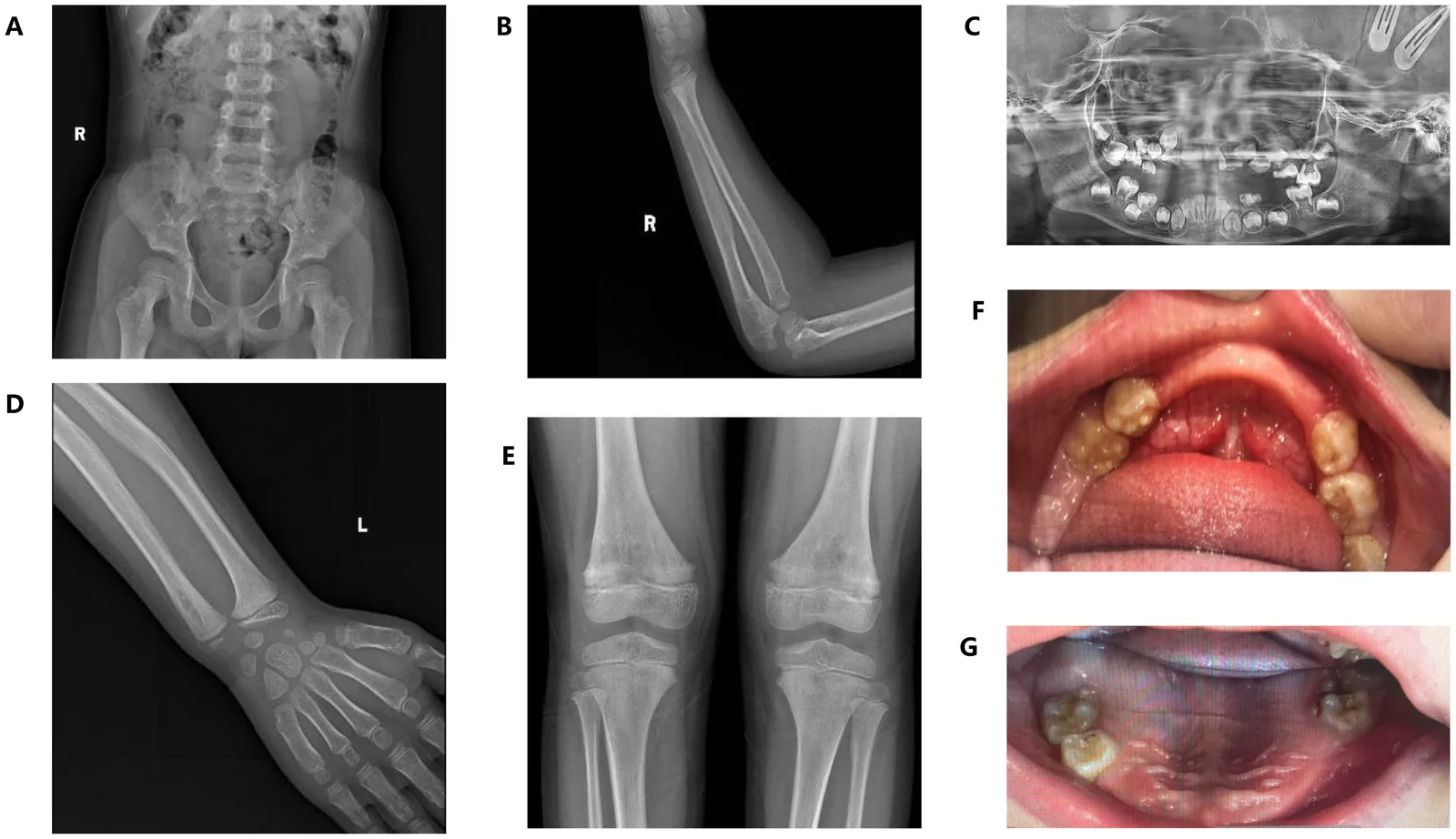
Skeletal and dental radiographs and oral photographs of PA-7. **(A)** shows mild wedge-like changes of several thoracic vertebrae, irregular margins of the sternum and distal sacrococcygeal region, cortical irregularity of the upper iliac margins, femoral heads and femoral necks, and generalized osteopenia. **(B, D)** show heterogeneous metaphyseal bone density in the distal radius and ulna with patchy radiolucent areas. **(E)** shows tongue-like metaphyseal changes of the distal femurs and patchy reductions in bone density around the distal femurs, proximal and distal tibiae/fibulae, epiphyses, and metaphyses. **(C)** shows multiple missing primary teeth and dental malalignment. **(F, G)** show upper and lower oral photographs.

PA-13 was a 10-year-old boy referred for bilateral lower-limb bowing for 9 years. He developed X-shaped legs, progressive out-toeing gait, and frontal bossing 9 years before presentation. Atraumatic primary tooth loss occurred at 16 months, followed by worsening gait abnormality and craniosynostosis surgery at our hospital. More than 1 year before the current visit, intermittent bilateral knee pain occurred every 2 weeks to 2 months.

At examination, height was 145 cm (75th percentile), weight was 58.5 kg (>97th percentile plus 8 kg), and BMI was 27.8 kg/m² (>95th percentile). He had obesity, cranial vault prominence, flat feet, scoliosis, genu valgum, and mild muscle weakness. Biochemistry showed low serum ALP (32 U/L) and low 25-hydroxyvitamin D (15 ng/mL), while serum calcium (2.51 mmol/L), phosphate (1.9 mmol/L), and urinary calcium/creatinine ratio were normal. Radiographs showed scoliosis, mild flattening of some lumbar vertebrae, distal radial bony protrusion/exostosis, and uneven density of the distal radius and ulna. Renal ultrasound showed no calcification. The diagnosis was revised to hypophosphatasia. This patient had the longest diagnostic delay in the cohort (9.1 years).

## 4 Discussion

This study systematically analyzed the clinical features, natural history, and ALPL variant spectrum of Chinese children with hypophosphatasia. The most clinically important finding was diagnostic delay in childhood hypophosphatasia: in the pooled cohort, the median age at onset was 1.13 years, whereas the median age at diagnosis was 4.00 years, corresponding to a median delay of 3.00 years. Infantile cases were often recognized earlier because of feeding difficulty, biochemical disturbances, and skeletal abnormalities. In contrast, childhood hypophosphatasia often began with early loss of primary teeth, mild skeletal deformity, abnormal gait, recurrent lower-limb pain, developmental bowing, or growth delay. These manifestations may be mistaken for isolated dental disease, vitamin D-deficiency rickets, or nonspecific growth problems (10, 12).

Patients with hypophosphatasia may be incorrectly treated for osteoporosis or rickets, and bisphosphonate therapy can aggravate impaired mineralization and increase fracture risk in hypophosphatasia (2, 15). The pediatric hypophosphatasia diagnostic criteria emphasize persistently low ALP interpreted using age- and sex-specific reference ranges (16). On this basis, early nontraumatic loss of primary teeth, the presence of rickets on radiographs, ALPL variants and elevated levels of natural TNSALP substrates are the most diagnostically valuable features (12). Among these, early-onset exfoliation of primary teeth is defined as the loss of primary teeth before 5 years of age, without obvious trauma and without root resorption (17). In the pooled cohort, early primary tooth loss occurred in 88.24% of patients with childhood hypophosphatasia and 100% of those with odontohypophosphatasia cases, and in the single-center cohort it was present in all childhood and odontohypophosphatasia patients.

HPP showed marked phenotypic heterogeneity, and earlier onset was associated with greater severity. In the pooled cohort, HAZ and WAZ were lowest in severe HPP and near normal in odonto-HPP. This pattern is consistent with previous pediatric natural history studies (17). Biochemically, severe HPP had lower ALP, phosphate, and PTH and higher serum calcium than childhood HPP and odonto-HPP, again consistent with the greater systemic severity of early-onset hypophosphatasia (17, 18). In the single-center cohort, onset before 6 months was characterized by failure to thrive (87.5%), delayed motor milestones (100%), nephrocalcinosis (100%), and hypercalcemia, whereas childhood hypophosphatasia was dominated by dental disease (100%), impaired mobility (100%), genu valgum/varum (66.67%), and chronic musculoskeletal pain (55.56%). This is consistent with findings reported in other studies (8). An observational analysis from the Global HPP Registry found that 81 children with disease onset before 6 months of age were more likely to exhibit skeletal deformities (59.3%), respiratory failure (38.3%), and hypercalcemia (44.4%), whereas 61 children with onset from 6 months to 18 years of age were more likely to exhibit premature loss of primary teeth (62.3%), delayed gross motor development (41%), and chronic bone pain (36.1%) (19).

Clinical classification of hypophosphatasia varies across publications. Recent literature describes multiple clinical forms, including perinatal, benign perinatal, infantile, mild childhood, severe childhood, adult, and odontohypophosphatasia (3, 20, 21). Using the conventional classification system, the cohort in the present study included 6 perinatal HPP, 28 infantile HPP, 34 childhood HPP, and 24 odonto-HPP. No benign prenatal cases were identified, which may be attributable to publication bias. As shown in **Supplementary Table 1**, perinatal and infantile HPP showed substantial overlap in major systemic manifestations and outcomes, whereas they were more clearly distinguishable from childhood HPP and odonto-HPP. Excessive subdivision therefore increases complexity without providing meaningful guidance for clinical management. These findings support the use of a simplified three-category framework for comparative analyses in this study: severe hypophosphatasia, defined as onset at ≤ 6 months; childhood hypophosphatasia, defined as onset from >6 months to <18 years with skeletal involvement; and odontohypophosphatasia, defined as dental manifestations without clear skeletal involvement.

Genetically, compound heterozygosity was the predominant genotype in Chinese children (78.57%), and variants were distributed across exons. The genetic pattern of HPP was similar in Chinese and Japanese cohorts, with compound heterozygous variants predominating (22),whereas Western pediatric cohorts showed a different pattern. Among 173 pediatric hypophosphatasia patients enrolled in a US study, genetic data were obtained for 105 cases, revealing 63 (60%) heterozygous and 42 (40%) compound heterozygous mutations (23). This difference may reflect variation in the patients enrolled in earlier studies and a greater proportion of US patients were diagnosed with mild childhood hypophosphatasia or odontohypophosphatasia. Severe disease has generally been associated with biallelic variants, whereas mild disease is more often associated with a single heterozygous variant (24). A genetic analysis of 98 unrelated Japanese patients found that the hotspot variant c.1559delT accounted for nearly half of all alleles (89/196, 45.4%) (22). The most frequent variant in our cohort was c.407G>A, but it accounted for only 7.14% of alleles, indicating that recurrent variants were present but did not dominate the Chinese pediatric spectrum.

Several observations suggest possible genotype-phenotype correlations. In this study, c.1101_1103delCTC (p.Ser368del) was identified only in severe hypophosphatasia, whereas c.1162T>C (p.Tyr388His) occurred only in childhood or odontohypophosphatasia. Recent structural work on TNSALP showed that the protein can form higher-order oligomeric assemblies and that pathogenic variants map to functional regions including the catalytic site, dimerization interface, and octamerization interface (25). S368 lies in a region involved in higher-order oligomeric stability, supporting a potential mechanistic link between p.Ser368del and severe disease. In contrast, p.Tyr388His has not been directly functionally validated, and further enzyme activity assays, protein stability studies, and segregation analyses are needed.

From the perspective of natural history, 3 of the 8 patients with infantile hypophosphatasia in the single-center cohort died of respiratory failure and 3 were lost to follow-up. This outcome is consistent with previous research showing a mortality rate as high as 50% among infants with hypophosphatasia who did not receive enzyme replacement therapy (1, 26). Individuals with perinatal onset of the condition face a high risk of mortality due to respiratory failure, respiratory distress, and vitamin B6-dependent epilepsy (6). However, PA-7 developed seizures during the neonatal period but remained clinically stable following symptomatic treatment and currently exhibits only dental malalignment and short stature. Follow-up of patients with childhood hypophosphatasia and odontohypophosphatasia in the single-center cohort showed progression in 4 children with childhood hypophosphatasia, while the remaining 5 were stable without improvement; all 6 children with odontohypophosphatasia remained stable. These findings are broadly consistent with the long-term follow-up of 101 children with hypophosphatasia (17), which showed that pediatric hypophosphatasia generally follows a chronic, persistent, and relatively stable course rather than undergoing clear spontaneous remission. However, the sample size of the follow-up in the present study is limited, and larger studies are warranted to substantiate this conclusion.

This study has limitations. First, the single-center cohort was small, and follow-up was partly retrospective and partly observational rather than strictly prospective; follow-up adherence varied. Second, literature cases came from different periods and institutions, and laboratory reference ranges, imaging protocols, and reporting completeness were inconsistent, which may have introduced selection and information bias. For instance, ALP assay methods and pediatric reference intervals were not fully standardized, and detailed laboratory reference ranges were unavailable in some reports. Therefore, differences in ALP levels across the pooled clinical subgroups may reflect both biological variation and inter-laboratory analytical variation and should be interpreted cautiously. Third, most patients lacked measurements of natural TNSALP substrates, such as pyridoxal 5’-phosphate, PPi, and phosphoethanolamine. Elevation of these substrates provides important supportive biochemical evidence for HPP, and their absence may elevate the risk of missed diagnoses. Multicenter prospective registry studies are needed to define the natural history of Chinese pediatric hypophosphatasia more accurately and to support earlier intervention strategies.

## 5 Conclusion

HPP is a continuous phenotypic spectrum involving bone, teeth, kidney, nervous system, and growth. Chinese pediatric hypophosphatasia showed marked clinical and genetic heterogeneity. Infantile hypophosphatasia had early onset, severe biochemical abnormalities, hypercalcemia, nephrocalcinosis, growth impairment, and poor outcomes. Childhood and odontohypophosphatasia often presented with early primary tooth loss, skeletal deformity, impaired mobility, and chronic musculoskeletal symptoms. Childhood hypophosphatasia was especially prone to missed or delayed diagnosis and did not show clear spontaneous improvement in this cohort. Atraumatic early loss of primary teeth, persistently low age- and sex-adjusted ALP, characteristic radiographic findings, and ALPL testing should be emphasized to improve early recognition. The ALPL variant spectrum in China was characterized by a predominance of missense variants and compound heterozygosity. Further large-scale multicenter and functional studies are warranted to better characterize genotype-phenotype correlations in Chinese children.

## Data Availability

The original contributions presented in the study are included in the article/**Supplementary Material**. Further inquiries can be directed to the corresponding author.
