# Supplementary material for: Natural history of hypophosphatasia in Chinese patients younger than 18 years: a single-center retrospective observational study
Source: Front Endocrinol (Lausanne). 2026 Sep 17;17:1941377. doi: 10.3389/fendo.2026.1941377 (PMC13626994; doi:10.3389/fendo.2026.1941377)
Supplement: Supplementary file 1 [file DataSheet1.pdf]

Table S1. Characteristics of pediatric patients with hypophosphatasia according to the conventional classification.

Variables	Total (n = 92)	Perinatal (n = 6)	Infantile (n = 28)	Childhood (n = 34)	Odonto (n = 24)
Sex, n (%)[a]					
Male	59 (64.13)	4 (66.67)	15 (53.57)	24 (70.59)	16 (66.67)
Female	32 (34.78)	1 (16.67)	13 (46.43)	10 (29.41)	8 (33.33)
Early loss of primary teeth, n (%)					
-	30 (32.61)	6 (100.00)	22 (78.57)	2 (5.88)	0 (0.00)
+	59 (64.13)	0 (0.00)	5 (17.86)	30 (88.24)	24 (100.00)
Short stature, n (%)					
-	47 (51.09)	5 (83.33)	8 (28.57)	14 (41.18)	20 (83.33)
+	35 (38.04)	1 (16.67)	19 (67.86)	13 (38.24)	2 (8.33)
Delayed motor milestones, n (%)					
-	30 (32.61)	6 (100.00)	4 (14.29)	4 (11.76)	16 (66.67)
+	32 (34.78)	0 (0.00)	20 (71.43)	12 (35.29)	0 (0.00)
Chronic musculoskeletal pain, n (%)					
-	54 (58.70)	6 (100.00)	20 (71.43)	9 (26.47)	19 (79.17)
+	15 (16.30)	0 (0.00)	2 (7.14)	13 (38.24)	0 (0.00)
Impaired mobility, n (%)					
-	25 (27.17)	5 (83.33)	2 (7.14)	1 (2.94)	17 (70.83)
+	37 (40.22)	1 (16.67)	19 (67.86)	17 (50.00)	0 (0.00)
Genu valgum/varum, n (%)					
-	45	6 (100.00)	16 (57.14)	7 (20.59)	16 (66.67)

Variables	Total (n = 92)	Perinatal (n = 6)	Infantile (n = 28)	Childhood (n = 34)	Odonto (n = 24)
	(48.91)				
+	15 (16.30)	0 (0.00)	5 (17.86)	10 (29.41)	0 (0.00)
Craniosynostosis, n (%)					
-	52 (56.52)	6 (100.00)	16 (57.14)	14 (41.18)	16 (66.67)
+	5 (5.43)	0 (0.00)	5 (17.86)	0 (0.00)	0 (0.00)
Failure to thrive, n (%)					
-	39 (42.39)	6 (100.00)	3 (10.71)	14 (41.18)	16 (66.67)
+	20 (21.74)	0 (0.00)	19 (67.86)	1 (2.94)	0 (0.00)
Recurrent fractures, n (%)					
-	53 (57.61)	5 (83.33)	19 (67.86)	10 (29.41)	19 (79.17)
+	12 (13.04)	1 (16.67)	3 (10.71)	8 (23.53)	0 (0.00)
Recurrent pneumonia, n (%)					
-	50 (54.35)	3 (50.00)	16 (57.14)	15 (44.12)	16 (66.67)
+	8 (8.70)	3 (50.00)	5 (17.86)	0 (0.00)	0 (0.00)
Hypotonia, n (%)					
-	34 (36.96)	2 (33.33)	7 (25.00)	9 (26.47)	16 (66.67)
+	27 (29.35)	3 (50.00)	16 (57.14)	7 (20.59)	1 (4.17)
Vitamin B6-responsive seizures, n (%)					
-	59 (64.13)	6 (100.00)	20 (71.43)	17 (50.00)	16 (66.67)
+	4 (4.35)	0 (0.00)	4 (14.29)	0 (0.00)	0 (0.00)
Nephrocalcinosis, n (%)e					
Not examined	58 (63.04)	5 (83.33)	11 (39.29)	25 (73.53)	17 (70.83)
-	16	1 (16.67)	1 (3.57)	7 (20.59)	7 (29.17)

Variables	Total (n = 92)	Perinatal (n = 6)	Infantile (n = 28)	Childhood (n = 34)	Odonto (n = 24)
	(17.39)				
+	18 (19.57)	0 (0.00)	16 (57.14)	2 (5.88)	0 (0.00)
Radiographic abnormalities, n (%)					
-	13 (14.13)	0 (0.00)	1 (3.57)	0 (0.00)	12 (50.00)
+	59 (64.13)	6 (100.00)	27 (96.43)	22 (64.71)	4 (16.67)
ALPL variant detected, n (%)					
Not examined	22 (23.91)	1 (16.67)	10 (35.71)	8 (23.53)	3 (12.50)
Positive	70 (76.09)	5 (83.33)	18 (64.29)	26 (76.47)	21 (87.50)
Genotype, n (%)	n = 70	n = 5	n = 18	n = 26	n = 21
Homozygous	4 (4.35)	0 (0.00)	2 (7.14)	2 (5.88)	0 (0.00)
Heterozygous	11 (11.96)	0 (0.00)	2 (7.14)	5 (14.71)	4 (16.67)
Compound heterozygous	55 (59.78)	5 (83.33)	14 (50.00)	19 (55.88)	17 (70.83)
Outcome, n (%)					
Lost to follow-up	42 (45.65)	1 (16.67)	9 (32.14)	20 (58.82)	12 (50.00)
Death	18 (19.57)	5 (83.33)	13 (46.43)	0 (0.00)	0 (0.00)
Alive	32 (34.78)	0 (0.00)	6 (21.43)	14 (41.18)	12 (50.00)

Abbreviations and notes: ALP, alkaline phosphatase; +, positive; -, negative. a, One perinatal lethal case had unspecified sex in literature review cohorts. Some data were missing; therefore, percentages were calculated using the observed number divided by the total number of patients in the relevant cohort or group.
